# Transition–Transversion Bias at the *CYTB* Gene Level in the Order Cypriniformes (Actinopterygii) as Evidence for the Influence of Metabolic Rate on Molecular Evolutionary Rate

**DOI:** 10.1002/ece3.73905

**Published:** 2026-06-29

**Authors:** S. V. Mezhzherin, S. Y. Morozov‐Leonov

**Affiliations:** ^1^ I. I. Schmalhausen Institute of Zoology Kyiv Ukraine

**Keywords:** body size, Cypriniformes, evolutionary rate hypothesis, metabolic rate, metabolic theory of ecology, molecular evolution, transition–transversion bias

## Abstract

A positive relationship between metabolic rate and the rate of mutation accumulation is regarded as a core prediction of the metabolic theory of ecology; however, empirical support for this relationship remains limited. One indirect but rigorous approach to testing this hypothesis is to analyze the transition–transversion bias across increasing levels of genetic divergence. This approach allows comparison of evolutionary substitution patterns among taxonomic groups that differ in metabolic rate, which can be inferred from body size and species‐specific body temperature. Because transitions largely arise from spontaneous molecular processes while transversions are more strongly associated with oxidative DNA damage. These two mutation types exhibit distinct origins and mutational spectra, comparison of which will allow us to estimate the rate of mutation caused by oxidative DNA damage in taxonomic groups with different metabolic rates. The order Cypriniformes represents an excellent model system for such analyses because it includes both small‐ and large‐bodied lineages distributed across tropical and temperate climatic zones. To identify patterns of evolutionary transition–transversion bias in the mitochondrial *CYTB* gene among cypriniform families and subfamilies differing in climatic affiliation and body size Families and subfamilies inhabiting temperate regions exhibit increasing transition frequencies and decreasing transversion frequencies with increasing genetic divergence, resulting in a pronounced transition–transversion bias. In contrast, tropical taxa showed elevated transversion frequencies and a substantially weaker bias. Taxa distributed across both climatic zones displayed intermediate characteristics. Large‐bodied fishes exhibited higher transition frequencies and lower transversion frequencies than small‐bodied taxa, resulting in a stronger transition–transversion bias. The greatest contrast was observed between small‐bodied tropical taxa and large‐bodied temperate taxa. The accelerated accumulation of transversions relative to transitions in taxa with higher metabolic intensity—small‐bodied fishes and tropical lineages—provides direct evidence for a positive relationship between metabolic rate and the rate of mutational processes, as well as a negative association between body size and mutation rate. Elevated transversion frequencies in tropical cypriniforms additionally support the evolutionary rate hypothesis, according to which warm and climatically stable environments promote accelerated molecular evolution.

## Graphical abstract

Families and subfamilies inhabiting temperate regions exhibit increasing transition frequencies and decreasing transversion frequencies with increasing genetic divergence, resulting in a pronounced transition–transversion bias. In contrast, tropical taxa show the opposite pattern, with elevated transversion frequencies and a markedly weaker bias. Taxa whose geographic ranges span both climatic zones display intermediate characteristics. Large‐bodied fishes show higher transition frequencies and lower transversion frequencies than small‐bodied taxa, leading to a greater magnitude of transition–transversion bias.

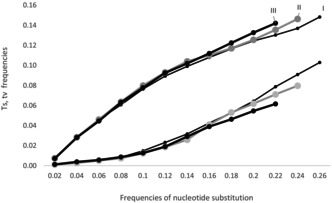

## 1 Introduction

The fundamental role of metabolism in shaping ecological and evolutionary processes has been formalized within the framework of the *metabolic theory of ecology* (Allen et al. 2002; Brown et al. 2004). This theory predicts that individual metabolic rate and environmental temperature influence speciation and extinction dynamics through their effects on generation time and mutation rate (Martin and Palumbi 1993; Gillooly et al. 2001, 2005, 2007; Gillooly and Allen 2007). A closely related concept is the *evolutionary rate hypothesis* (Rensch 1959), which proposes that the relatively constant high temperatures characteristic of tropical climates accelerate biological processes, leading to shorter generation times, higher mutation rates, and faster evolutionary change. Together, these mechanisms have been invoked to explain elevated speciation rates and the pronounced latitudinal diversity gradient observed across many taxonomic groups (Rohde 1992).

Despite the conceptual appeal of these hypotheses, empirical evidence linking individual metabolic rate to the rate of mutational processes remains inconsistent (Nabholz, Glémin, et al. 2008; Galtier et al. 2009). Experimental studies demonstrating increased mutation output under elevated temperatures in laboratory organisms (Lindgren 1972; Chu et al. 2018) may not accurately reflect mutation dynamics under natural conditions. Similarly, evidence for a relationship between body size and genetic variability is equivocal. Some studies report a negative association between nucleotide diversity and mass‐specific metabolic rate at the species level (James and Eyre‐Walker 2020), whereas others fail to detect such a pattern (Nabholz, Mauffrey, et al. 2008; Clark et al. 2023). Comparisons of molecular evolutionary rates between tropical and temperate regions are likewise contradictory, with studies variously supporting (Oppold et al. 2016; Dowle et al. 2013), partially supporting (Gillman and Wright 2013; Cai et al. 2025), or rejecting (Liu et al. 2023) the evolutionary rate hypothesis.

A major obstacle to resolving these discrepancies is the difficulty of estimating absolute mutation rates across broad taxonomic scales. This challenge arises mainly from the scarcity of robust paleontological calibrations. Relative measures of molecular evolution therefore offer a promising alternative. One such measure is the transition–transversion bias (Li and Graur 1991). Under spontaneous mutational processes, transition substitutions (purine–purine or pyrimidine–pyrimidine) occur far more frequently than transversions (purine–pyrimidine), despite the greater number of possible transversion changes (Fitch 1967; Vogel and Kopun 1977; Jukes 1987). In vertebrates, this bias is pronounced at intraspecific and interspecific levels of divergence and diminishes at the family level. At higher taxonomic levels, the pattern may reverse, with transversions predominating and substitution patterns approaching random expectations (Rosenberg et al. 2003; Stoltzfus and Norris 2016; Mezhzherin et al. 2024).

Mechanistically, transitions are thought to arise primarily from DNA tautomerism (Löwdin 1966), whereas transversions are largely attributable to oxidative DNA damage (Shigenaga et al. 1989). Oxidative DNA damage increases with cellular oxygen saturation and, therefore, with metabolic intensity. Consequently, an elevated metabolic rate should lead to disproportionately higher transversion frequencies and can serve as a useful indicator of physiological and evolutionary processes (Iliushchenko et al. 2025). As a result, the magnitude of the transition–transversion bias is expected to decline with increasing metabolic rate. Comparative analyses of transition and transversion accumulation across taxonomic groups that differ in metabolic intensity, therefore, provide a powerful framework for testing predictions of metabolic theory.

Ray‐finned fishes represent an especially suitable model for such analyses because their metabolic rates are influenced not only by intrinsic factors such as evolutionary history and body size, but also by external environmental variables including temperature, dissolved oxygen concentration, and salinity (Gillooly et al. 2001; White et al. 2006; Chabot et al. 2016; Enders and Boisclair 2016). Within this group, the order Cypriniformes is particularly well suited for comparative study. It comprises approximately 5000 freshwater species distributed among at least 12 families and four suborders and represents a key lineage for investigation of vertebrate evolution in freshwater ecosystems (Sudasinghe et al. 2026). The order includes both tropical and strictly temperate lineages, spans a wide range of body sizes, and is characterized by extensive mitochondrial *CYTB* gene investigations (Rüber et al. 2007; Alam et al. 2021; Chen et al. 2025).

These characteristics make Cypriniformes an ideal system for examining evolutionary transition–transversion bias along two major axes that shape metabolic rate: environmental temperature and body size. By integrating these dimensions, the present study aims to provide a rigorous empirical test of the relationship between metabolic intensity and mutation rate, and thereby to evaluate key predictions of the metabolic theory of ecology.

## 2 Materials and Methods

### 2.1 Data Sampling and Taxonomic Coverage

Evolutionary transition–transversion bias was analyzed by comparing substitution patterns across taxonomic groups of suborder, family, and subfamily rank within the order Cypriniformes on the basis of sequences of mitochondrial cytochrome *b* ( *CYTB* ) gene. This single‐copy gene, characterized by a relatively high level of variability and a comparatively long nucleotide sequence, is widely used as a molecular marker in vertebrate evolutionary studies (Weir and Schluter 2008; Rahmadina and Tjong 2024). Consequently, it is frequently employed in large‐scale comparative analyses involving hundreds or thousands of taxa (Johns and Avise 1998; Kartavtsev 2011). The gene has also proven useful in studies of evolutionary transition–transversion dynamics (Yang and Yoder 1999; Mezhzherin and Morozov‐Leonov 2024). Furthermore, *CYTB* is the most extensively sequenced gene in Cypriniformes, enabling inclusion of thousands of species and virtually all family‐level taxonomic groups in the present study. Ultimately, the analysis of this gene's variability enables a rigorous comparative study and allows for a degree of extrapolation to broader genomic variability.

For the most species‐rich families (Cyprinidae, Leuciscidae, and Danionidae), analyses were conducted at the subfamily level to ensure adequate taxonomic resolution (Table S1). In total, 29 taxonomic groups were included in the study.

The dataset consisted of mitochondrial *CYTB* sequences retrieved from GenBank in spring 2025. Most sequences were complete (1140–1143 b.p.); in exceptional cases, partial sequences of at least 900 bp were used. In total, 1705 sequences were analyzed. These records consisted primarily of individual *CYTB* sequences and, less frequently, complete mitochondrial genome fragments. Unverified sequences were excluded. Sequence quality was assessed by calculating genetic distances and constructing distance matrices and phenograms, allowing identification of anomalous sequences, the number of which proved negligible.

### 2.2 Biogeographic Classification

Based on the modern global map of terrestrial ecoregions (Olson et al. 2001), families and subfamilies of Cypriniformes were assigned to one of three bioclimatic zones (Table S1).

Tropical group (I): families and subfamilies confined to tropical climatic zones with consistently warm environments and distributed primarily within the Indomalayan and Afrotropical realms.

Mixed‐zone group (II): families and subfamilies composed predominantly of Indomalayan taxa but including species with broader Palearctic distributions.

Temperate group (III): families and subfamilies characteristic of strongly seasonal climatic zones and restricted to the Holarctic realm (Palearctic and/or Nearctic).

### 2.3 Body‐Size Classification

Data on species body‐size characteristics were obtained from FishBase (2025). Because these data are incomplete, maximum body length and body mass values were used instead of mean values. Maximum body length information was available for all analyzed taxa, allowing direct comparisons among families and subfamilies (Table S1).

Suborders and families/subfamilies were classified as two body size categories.

The small‐bodied group included taxa whose representatives reached a maximum body mass of up to 1000 g and a standard body length of up to 40 cm. The large‐bodied group included taxa whose representatives attained a maximum body mass of at least 1500 g and a body length of at least 50 cm.

At the suborder level, the small‐bodied group included Gyrinocheiloidei and Cobitoidei, whose representatives generally did not exceed 1000 g in body mass, whereas the large‐bodied group consisted of Catostomoidei and Cyprinoidei.

### 2.4 Estimation of Transition–Transversion Bias

The magnitude and structure of evolutionary transition–transversion bias were assessed at the family and subfamily levels using the approach proposed by Yang and Yoder (1999). This method evaluates changes in transition and transversion frequencies, as well as their ratios, across increasing classes of genetic divergence. The authors estimated transition/transversion rate ratios through pairwise sequence comparisons and joint likelihood analyses using mitochondrial cytochrome *b* genes. This approach minimizes the influence of taxonomic uncertainty.

In direct pairwise comparisons of aligned sequences, the summed frequencies of homotypic substitutions (purine ↔ purine and pyrimidine ↔ pyrimidine) were used as estimates of transition frequency. Correspondingly, the summed frequencies of heterotypic substitutions (purine ↔ pyrimidine and *vice versa*) were used to estimate transversion frequency.

Differences in the accumulation of transitions and transversions, as well as variation in transition–transversion bias among taxonomic groups differing in body size and climatic affiliation, were evaluated using two complementary approaches.

The first approach involved pairwise sequence comparisons to obtain mean transition and transversion frequencies together with the transition–transversion index across nucleotide‐substitution classes. According to previously established scaling of nucleotide substitutions in Palearctic Murinae based on the *CYTB* gene (Mezhzherin and Tereshchenko 2023), nucleotide substitutions were divided into classes with intervals of 0–0.02. Under this framework, 0–0.02 corresponds to individual and interpopulation variation; 0.02–0.04 corresponds to interpopulation divergence and the earliest stages of speciation; 0.04–0.08 corresponds to semispecies divergence; 0.08–0.16 corresponds to species‐level divergence; and 0.14–0.32 corresponds to divergence among species belonging to different genera. For each substitution class, average transition and transversion frequencies were calculated, followed by evaluation of statistical significance using Student's *t*‐test.

The second approach involved estimation of average rates of change in transition and transversion frequencies, as well as in the transition–transversion index, across divergence classes using two‐factor ANOVA. In these analyses, the first factor represented substitution characteristics in different geographic or body‐size groups, whereas the second factor represented classes of nucleotide divergence.

The magnitude of transition–transversion bias was estimated using the index *K* = (ts − tv)/(ts + tv), where ts and tv represent transition and transversion frequencies, respectively. This index ranges from 1 (transversion frequency equals zero) to −1 (transition frequency equals zero) and equals zero when transition and transversion frequencies are identical. Compared with the traditional ts/tv ratio, this index allows a consistent assessment of substitution bias even when transversion frequencies approach zero.

### 2.5 Sequence Alignment and Statistical Analyses

Sequence alignment was performed using BioEdit v7.2.5 with the ClustalW algorithm (full multiple alignment, bootstrap NJ tree, 1000 bootstraps) (Abbott Laboratories, Green Oaks, Illinois, USA, 1999) (Hall 1999). Pairwise genetic distances, distance matrices, and phenograms were generated in MEGA v11.0.11 (Institute of Molecular Evolutionary Genetics, The Pennsylvania State University, University Park, Pennsylvania, USA, 2021) (Tamura et al. 2021) to verify sequence quality and taxonomic consistency. Frequencies of different nucleotide substitution types were calculated directly from pairwise sequence comparisons.

Importantly, all pairwise comparisons were performed within each taxonomic group separately, and subsequent analyses of transition/transversion dynamics were conducted within divergence classes, not across species as independent units. Consequently, the statistical framework does not rely on species‐level trait independence, and phylogenetic correction methods (e.g., PGLS or independent contrasts) are neither required nor applicable to the presented comparisons.

## 3 Results

Mean transition and transversion frequencies, together with mean transition–transversion index values across nucleotide‐substitution classes for each family‐ and subfamily‐level taxonomic group, are presented in Table S2.

### 3.1 Comparisons Among Taxonomic Groups From Different Bioclimatic Zones

**FIGURE 1:**
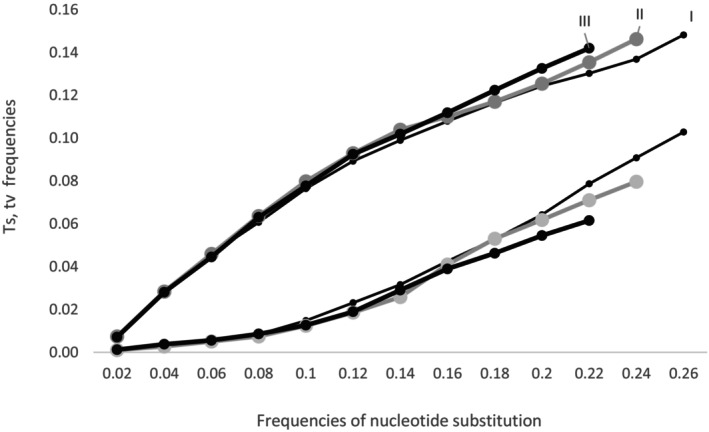
Trends of transition (top lines) and transversion (bottom lines) mean frequency changes per classes of nucleotide substitutions of *CYTB* within family/subfamily groups of the Cypriniformes with ranges of tropical (I), mixed (II), and temperate (III) zones.

**FIGURE 2:**
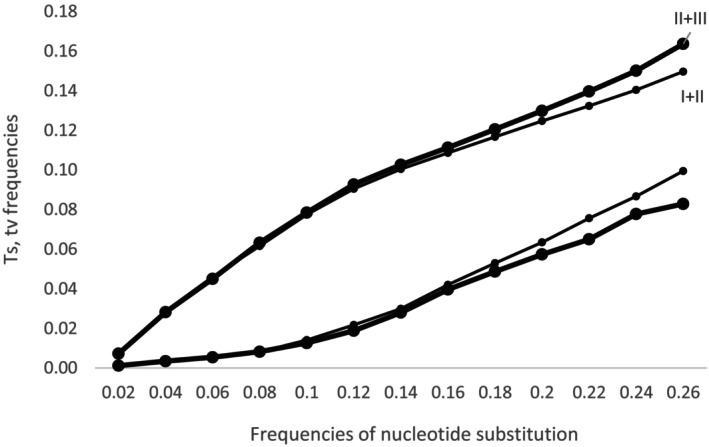
Trends of transition (top lines) and transversion (bottom lines) mean frequencies changes per classes of nucleotide substitutions of *CYTB* within family/subfamily groups of the Cypriniformes of tropical + mixed (I + II) and mixed + temperate (II + III) zones.

Patterns of transition and transversion frequencies across frequencies of nucleotide substitution for families and subfamilies representing different bioclimatic zones are shown in Figures 1 and 2.

Across all five comparison schemes, a consistent general pattern emerged.

### 3.2 Tropical Versus Mixed‐Zone Taxa

Transition frequencies were generally lower in tropical families and subfamilies (Table S3). This pattern was supported both by significant differences in mean values across four nucleotide‐substitution classes (Table S4) and by differences in the mean rate of transition accumulation across divergence classes (*F* = 7.2; df_1_ = 1; df_2_ = 156; *p* < 0.01). In contrast, transversion frequencies exhibit the opposite trend (Table S5): means are significantly higher in tropical taxa in two nucleotide substitution classes (Table S6), and differences in the mean rates of accumulation are highly significant (*F* = 14.9; df_1_ = 1; df_2_ = 156; *p* < 0.001). Consequently, the magnitude of the transition–transversion bias is reduced in tropical taxa (Table S7), as reflected both in significantly lower mean index values in four comparisons (Table S8) and in differences in the mean rates of index change (*F* = 7.7; df_1_ = 1; df_2_ = 156; *p* < 0.01).

### 3.3 Tropical Versus Temperate Zone

A similar but more pronounced pattern was observed (Table S3). Transition frequencies are lower in tropical families and subfamilies, with significant differences in two substitution classes and significant differences in the mean rates of accumulation process (*F* = 15.1; df_1_ = 1; df_2_ = 198; *p* < 0.001) (Table S4). Transversion frequencies are higher in tropical taxa in three substitution classes (Table S5), with highly significant differences in the mean rates of accumulation (*F* = 25.9; df_1_ = 1; df_2_ = 198; *p* < 0.001) (Table S6). Accordingly, mean transition–transversion index values are lower in tropical taxa in three comparisons but higher in one (Table S7), and differences in the mean rates of index change are significant (*F* = 8.0; df_1_ = 1; df_2_ = 198; *p* < 0.001) (Table S8).

### 3.4 Mixed Versus Temperate Zone

Differences in mean transition frequencies between mixed‐zone and temperate families are significant in two comparisons (Table S4), but mean rates of transition accumulations do not differ (*F* = 2.8; df_1_ = 1; df_2_ = 134; *p* > 0.05) (Table S4). However, transversion frequencies are higher in mixed‐zone taxa in three nucleotide substitution classes, accompanied by significant differences in the mean rates of accumulation (*F* = 4.6; df_1_ = 1; df_2_ = 134; *p* < 0.05) (Tables S5 and S6). Transition–transversion index values are lower in mixed‐zone taxa in three comparisons, although differences in the mean rates of index change are not significant (*F* = 0.02; df_1_ = 1; df_2_ = 134; *p* > 0.05) (Tables S7 and S8).

### 3.5 Tropical and Mixed Zones Combined Versus Temperate Zone

When tropical and mixed‐zone taxa are jointly compared with temperate taxa, the analogical pattern is evident, although less pronounced (Tables S3–, S8). Transition frequencies are lower in taxa with tropical representation in four comparisons, with a reduced mean rate of accumulation (*F* = 16.5; df_1_ = 1; df_2_ = 252; *p* < 0.001) (Table S4). Transversion frequencies are higher in four comparisons, with a significantly higher mean rate of accumulation (*F* = 25.5; df_1_ = 1; df_2_ = 252; *p* < 0.001) (Table S6). Transition–transversion index values show a decreasing trend in tropical and mixed‐zone taxa, supported by four significant pairwise comparisons and a significant difference in mean rates of index change (*F* = 5.0; df_1_ = 1; df_2_ = 252; *p* < 0.05) (Table S8).

### 3.6 Tropical Versus Mixed and Temperate Zones Combined

The general trend persists when tropical taxa are compared with all non‐tropical taxa combined (Tables S3–, S8). Mean transition frequencies are lower in tropical groups, with significant differences observed in two comparisons, as well as a lower mean rate of accumulation (*F* = 13.2; df_1_ = 1; df_2_ = 252; *p* < 0.001) (Table S4). In contrast, mean transversion frequencies are higher in tropical taxa in four comparisons and exhibit a substantially higher rate of accumulation (*F* = 25.5, df_1_ = 1, df_2_ = 252, *p* < 0.001). Accordingly, transition–transversion index values are lower in tropical taxa, with significant differences detected in four comparisons and in the mean rate of index change (*F* = 10.8, df_1_ = 1, df_2_ = 252; *p* < 0.001) (Table S8).

### 3.7 Comparison of Small and Large Body‐Sized Taxonomic Groups

Comparisons between small‐ and large‐bodied taxa were conducted at two taxonomic levels: (i) the suborder level, contrasting the small‐bodied Gyrinocheiloidei and Cobitoidei with the large‐bodied Cyprinoidei and Catostomoidei; and (ii) the family/subfamily level (Table S1).

**FIGURE 3:**
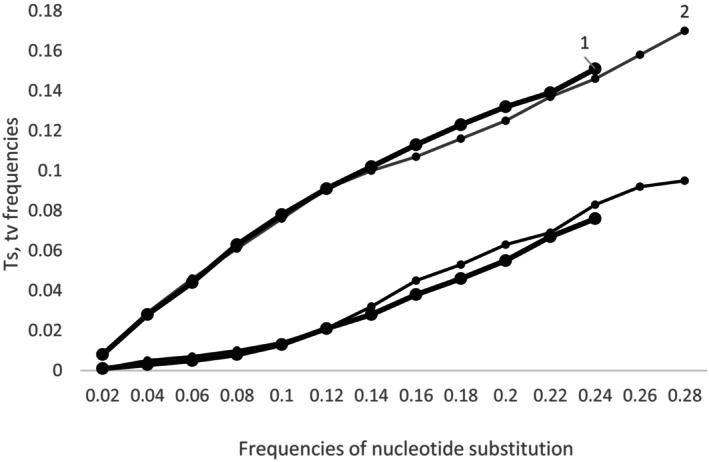
Trends of transition (top lines) and transversion (bottom lines) mean frequencies changes of different classes of nucleotide substitutions of *CYTB* within small body sized (1) and large body sized (2) of *Cypriniformes* families/subfamilies.

**FIGURE 4:**
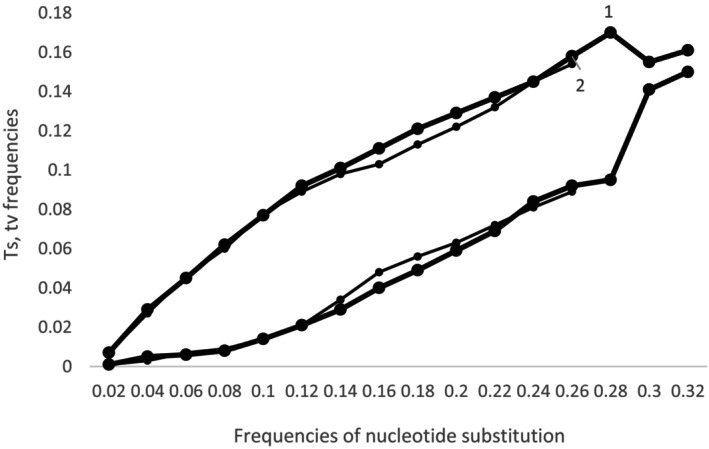
Trends of changes in transition (ts) and transversion (tv) average frequencies in different classes of the nucleotide substitutions of *CYTB* within large body sized (1) and small body sized (2) of the *Cypriniformes* suborders.

Mean transition frequencies are generally higher in large‐bodied families and suborders than in small‐bodied taxa (Figures 3 and 4; Tables S9 and S10). This pattern is reflected both in significant pairwise differences among individual substitution classes and in higher mean rates of transition accumulation across divergence classes at the family/subfamily (*F* = 16.4, df_1_ = 1, df_2_ = 259, *p* < 0.001) (Table S9) and suborder (*F* = 11.8, df_1_ = 1, df_2_ = 252, *p* < 0.001) (Table S10) levels.

In contrast, transversion frequencies exhibit the opposite trend (Figures 3 and 4; Tables S9 and S10). Small‐bodied taxa show higher transversion frequencies, a pattern consistently supported by pairwise comparisons and by significant differences in the mean rates of transversion accumulation at both the family/subfamily (*F* = 21.8, df_1_ = 1, df_2_ = 259, *p* < 0.001) (Table S9) and suborder (*F* = 9.4, df_1_ = 1, df_2_ = 252, *p* < 0.01) (Table S10) levels.

These contrasting substitution dynamics result in pronounced differences in the magnitude of transition–transversion bias (Tables S9 and S10). On average, large‐bodied taxa exhibit a greater degree of transition–transversion bias than small‐bodied taxa. This trend is consistently supported both by pairwise comparisons and by differences in the mean rates of change in the transition–transversion index across divergence classes at the family/subfamily (*F* = 24.4, df_1_ = 1, df_2_ = 259, *p* < 0.001) (Table S9) and suborder (*F* = 6.8, df_1_ = 1, df_2_ = 252, *p* < 0.05) (Table S10) levels.

### 3.8 Combined Effects of Body Size and Climatic Affiliation

**FIGURE 5:**
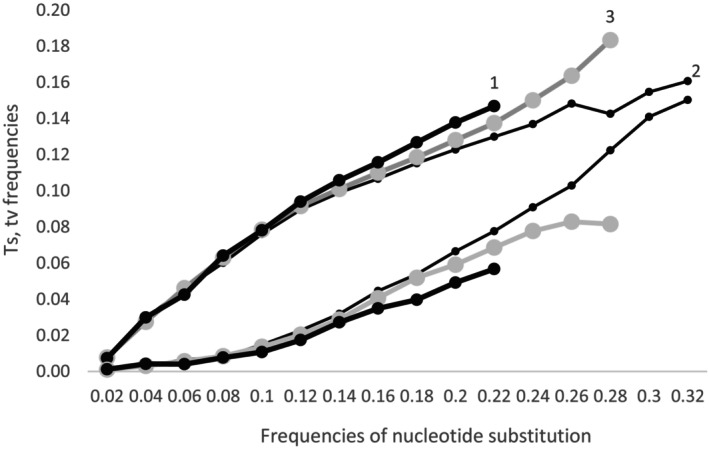
Trends of transition (top lines) and transversion (bottom lines) mean frequencies changes of different classes of the nucleotide substitutions of *CYTB*: 1—group C (large body sized temperate families); 2—group A (small body sized tropical families); 3—group B (families with intermediate parameters).

To assess the combined effects of body size and climatic affiliation, taxa were grouped into three categories: (group A) small body‐sized families from tropical bioclimatic zone; (group B) small body‐sized taxa from the temperate zone, large body‐sized taxa from tropical zone as well as families with ranges occupying temperate and tropical zones; (group C) large body‐sized families of temperate zone. Comparisons reveal decreasing transition frequencies and increasing transversion frequencies in group A relative to group B and group C, as well as group B to group C (Figure 5). As a result, the maximum level of transition–transversion index variation is found in group C in comparison with group A (*F* = 21.1; df_1_ = 1; df_2_ = 106; *p* < 0.001) and group B (*F* = 13.9; df_1_ = 1; df_2_ = 163; *p* < 0.001), and the minimum level in group A (Table S11). This observation suggests that the greatest differences in patterns of nucleotide variability occur between groups representing opposite extremes of metabolic rate, namely small‐bodied families from tropical regions and large‐bodied families from temperate regions.

## 4 Discussion

The results of this study provide strong support for two major evolutionary hypotheses.

First, the magnitude of transition–transversion bias decreases systematically from temperate to tropical cypriniform lineages. This pattern is driven primarily by accelerated accumulation of transversions in tropical taxa, whereas changes in transition frequencies are comparatively modest. The most parsimonious explanation for the observed reduction in transition–transversion bias is therefore not suppression of transitions, but rather disproportionate acceleration of transversion accumulation. These findings indicate that variation in transversion rates is the principal determinant of evolutionary transition–transversion bias in Cypriniformes.

Second, a clear association exists between body‐size characteristics and patterns of transition–transversion bias at both suborder and family/subfamily levels. Large‐bodied taxa consistently exhibited higher transition frequencies and lower transversion frequencies than small‐bodied taxa, resulting in stronger transition–transversion bias. These patterns were robust across multiple analytical approaches and divergence classes.

Importantly, climatic affiliation and body size do not exhibit a simple one‐to‐one correspondence at the family or subfamily level. Tropical regions include both some of the smallest‐bodied cypriniform lineages (e.g., Paedocyprididae, Tanichthyidae, and Gastromyzontidae) and relatively large‐bodied taxa (e.g., Labeoninae). Conversely, temperate families and subfamilies encompass a broad range of body sizes, including relatively small‐bodied taxa (e.g., Pogonichthyinae, Gobionidae, and Cobitidae) as well as comparatively small representatives within nominally large‐bodied groups (e.g., Leuciscinae and Pseudaspininae).

This heterogeneity suggests that body size and environmental temperature influence substitution rates and the structure of transition–transversion bias both independently and interactively. The strongest manifestation of these combined effects is observed in large‐bodied Holarctic taxa (e.g., Catostomidae, Leuciscinae, Pseudaspininae, and Plagopterinae), which exhibit the highest levels of transition–transversion bias.

Taken together, these findings provide a coherent and empirically grounded test of a central prediction of the metabolic theory of ecology (Allen et al. 2002; Brown et al. 2004). In ectothermic organisms such as cypriniform fishes, individual metabolic rate is determined primarily by body size and environmental temperature (Gillooly et al. 2001; White et al. 2006; Chabot et al. 2016; Enders and Boisclair 2016). Accordingly, the highest metabolic intensities are expected in small‐bodied tropical taxa, whereas the lowest should characterize large‐bodied temperate endemics. These metabolic differences should be reflected in mutation dynamics, with elevated transversion accumulation in high‐metabolism taxa leading to a reduced transition–transversion bias. The results of the present study are broadly consistent with this prediction. At the group level, they provide evidence for a positive relationship between metabolic rate and mutation rate, as manifested through substitution spectra rather than absolute substitution counts.

The conclusions of this study are based on relative differences in transition and transversion rates and are therefore inherently indirect. Ideally, analyses of this kind should be based on estimates of absolute substitution rates, which would require robust paleontological age calibrations for the taxa under consideration, at least at the family level. However, given the current state of knowledge, relatively reliable age estimates appear to be available only at the level of actinopterygian orders (Benton 2014).

The demonstration of a positive relationship between metabolic rate and mutation rate in the *CYTB* gene, based on comparisons among families within the order Cypriniformes, raises the question of whether this trend can be extrapolated to the genome as a whole. There are grounds to assume that results obtained for a gene that has proven to be a reliable marker of evolutionary processes may be applicable to broader patterns of genomic variability. However, it should be emphasized that not all genes exhibit differences in transition and transversion rates (Keller et al. 2007).

Analyses of multiple mitochondrial and nuclear DNA sequences in the Palearctic subfamily Murinae (Mezhzherin et al. 2023) demonstrated that differences in transition and transversion rates are characteristic primarily of hypervariable and highly variable DNA markers. In the hypervariable D‐loop region, complete compensation of transition–transversion bias occurs already at the level of species divergence. In the highly variable genes *CYTB* and *COX1*, equalization of transition and transversion frequencies is observed only at the level of divergence between distantly related genera. By contrast, conservative genes such as *12S rRNA, IRBP*, and *Fv* do not exhibit differences in the rates of transition and transversion accumulation within families, and the observed decrease in the transition/transversion ratio across evolutionary series appears to be largely mathematical in nature.

Therefore, the results obtained for the *CYTB* gene can likely be extrapolated only to the hypervariable and rapidly evolving fraction of the genome, whereas the applicability of these patterns to conserved structural genes remains unresolved.

The present study also highlights several auxiliary yet theoretically important patterns. One of these concerns the long‐standing hypothesis of a negative relationship between body size and mutation rate. Although such a relationship is predicted by metabolic theory (Gillooly et al. 2001), empirical support has remained limited and, in some cases, contradictory (Nabholz, Glémin, et al. 2008; Nabholz, Mauffrey, et al. 2008).

Evidence derived from taxonomic differentiation among large‐bodied vertebrates is often affected by biases associated with differential morphological diagnosability, potentially leading to underestimation of genetic divergence in large‐bodied taxa (Mezhzherin and Morozov‐Leonov 1996). Similarly, population‐level data provide only indirect evidence, because reduced genetic polymorphism in large‐bodied species is frequently attributable to smaller effective population sizes rather than to lower mutation rates themselves (Mezhzherin 2002).

In this context, the consistently higher rates of transversion accumulation observed in small‐bodied Cypriniformes, together with similar patterns recently reported in birds and mammals (Mezhzherin and Morozov‐Leonov 2024), provide strong evidence for a genuine negative association between body size and mutation rate.

Another important implication of the present study concerns the evolutionary rate hypothesis, specifically the prediction that warm, thermally stable environments promote elevated mutation rates and, potentially, increased rates of speciation (Allen et al. 2002, 2006; Gillooly et al. 2001, 2007; Gillooly and Allen 2007). This hypothesis is supported by the higher transversion frequencies observed in tropical cypriniform families and subfamilies.

Importantly, the acceleration demonstrated here primarily involves transversions. This does not exclude the possibility that transition rates also increase under tropical conditions; rather, it suggests that the magnitude of acceleration is substantially greater for transversions. Consequently, the overall transition–transversion bias decreases even if both classes of substitutions increase in absolute terms.

The molecular evidence supporting the evolutionary rate hypothesis presented here is particularly significant in light of ongoing debates concerning the causes of the latitudinal diversity gradient. Although the elevated species richness of tropical regions has often been interpreted as indirect evidence of accelerated evolutionary rates, such arguments are weakened by the presence of similar diversity gradients in both ectothermic and endothermic taxa, implying an important contribution of historical and ecological factors. In contrast, the present results demonstrate a positive relationship between metabolic intensity and mutational output at the molecular level. They indicate that the high and thermally stable conditions characteristic of tropical environments contribute to increased rates of molecular evolution—at least in ectothermic organisms—by promoting the accumulation of metabolically mediated mutational changes.

## Author Contributions

**S. V. Mezhzherin:** conceptualization (lead), formal analysis (equal), investigation (equal), methodology (equal), project administration (equal), validation (equal), visualization (equal), writing – original draft (equal), writing – review and editing (equal). **S. Y. Morozov‐Leonov:** conceptualization (supporting), formal analysis (equal), investigation (equal), methodology (supporting), writing – original draft (equal), writing – review and editing (equal).

## Funding

This study was financially supported within the framework of planned budget funding from the National Academy of Sciences of Ukraine (planned project no. III‐55‐21).

## Ethics Statement

The collection of materials for this study was carried out in full compliance with the national and European laws on bioethics.

## Conflicts of Interest

The authors declare no conflicts of interest.

## Supporting information


**Table S1:** ece373905‐sup‐0001‐TableS1.xlsx.

**Table S2:** ece373905‐sup‐0002‐TableS2.xlsx.

**Table S3:** Means (M) and standard errors (SE) of transition frequencies within classes of nucleotide substitutions and number of subfamilies/families (N) within bioclimatic zones.

**Table S4:** Student's t‐tests (t) and number of degrees of freedom (df) as well results of two‐way ANOVA (*F*) obtained from comparisons of mean transition frequencies of nucleotide substitution classes of Cypriniformes subfamilies/families of different bioclimatic zones.

**Table S5:** Means (M) and standard errors (SE) of transversion frequencies within classes of nucleotide substitutions and number of subfamilies/families (N) within bioclimatic zones.

**Table S6:** Student's t‐tests (t) and number of degrees of freedom (df) as well results of two‐way ANOVA (*F*) obtained from comparisons of mean transversion frequencies of nucleotide substitution classes of Cypriniformes subfamilies/families of different bioclimatic zones.

**Table S7:** Means (M) and standard errors (SE) of ts/tv index within classes of nucleotide substitutions and number of subfamilies/families (N) within bioclimatic zones.

**Table S8:** Student's t‐tests (t) and number of degree of freedoms (df) as well results of two‐way ANOVA (*F*) obtained from comparisons of mean transversion frequencies of nucleotide substitution classes of Cypriniformes subfamilies/families of different bioclimatic zones.

**Table S9:** Means (M), standard errors (SE) of transition (Ts), transversion (Tv) frequencies, ts/tv indices (Ts/Tv) and number of subfamilies/families (N) for each class of nucleotide substitutions in small body sized and large body sized subfamilies/families of the Cypriniformes and its comparisons by Student's t‐test and two‐way ANOVA.

**Table S10:** Means (M). standard errors (SE). of transition (Ts). transversion (Tv) frequencies. ts/tv indices (Ts/Tv) and number of subfamilies/families (N) for each class of nucleotide substitutions in small‐body sized and large‐body sized suborders of the Cypriniformes and its comparisons by Student's t‐test and two‐way ANOVA.

**Table S11:** Means (M) and standard errors (SE) of ts/tv index within classes of nucleotide substitutions and number of analyzed of subfamilies/families (N) of climatic zone types and body sized groups combined and its comparisons by Student's t‐test and two‐way ANOVA.

## Data Availability

The data supporting the findings of this study are available in the Supporting Information deposited at Figshare: https://doi.org/10.6084/m9.figshare.32507679.
