# Supplementary material for: Transition–Transversion Bias at the CYTB Gene Level in the Order Cypriniformes (Actinopterygii) as Evidence for the Influence of Metabolic Rate on Molecular Evolutionary Rate
Source: Ecol Evol. 2026 Jun 29;16(7):e73905. doi: 10.1002/ece3.73905 (PMC13314720; doi:10.1002/ece3.73905)
Supplement: Supplementary file 3 — Table S3: Means (M) and standard errors (SE) of transition frequencies within classes of nucleotide substitutions and number of subfamilies/families (N) within bioclimatic zones. [file ECE3-16-e73905-s010.docx]

Table S3. Means (M) and standard errors (SE) of transition frequencies within classes of nucleotide substitutions and number of subfamilies/families (N) within bioclimatic zones

| Substitu-tion  classes | Bioclimatic zones | | | | | | | | | | | | |
| --- | --- | --- | --- | --- | --- | --- | --- | --- | --- | --- | --- | --- | --- |
|  | I | | | II | | | III | | | I + II | | II + III | |
|  | M | SE | N | M | SE | N | M | SE | N | M | SE | M | SE |
| 0-0.02 | 0.007 | 0.0010 | 13 | 0.007 | 0.0008 | 5 | 0.007 | 0.0007 | 10 | 0.007 | 0.0008 | 0.007 | 0.0005 |
| 0.02-0.04 | 0.029 | 0.0028 | 10 | 0.028 | 0.0011 | 5 | 0.028 | 0.0010 | 10 | 0.029 | 0.0019 | 0.028 | 0.0008 |
| 0.04-0.06 | 0.046 | 0.0018 | 13 | 0.046 | 0.0009 | 5 | 0.045 | 0.0010 | 9 | 0.046 | 0.0013 | 0.045 | 0.0007 |
| 0.06-0.08 | 0.061 | 0.0010 | 12 | 0.064 | 0.0011 | 5 | 0.063 | 0.0008 | 10 | 0.062 | 0.0008 | 0.063 | 0.0006 |
| 0.08-0.10 | 0.076 | 0.0010 | 12 | 0.080 | 0.0007 | 5 | 0.078 | 0.0008 | 10 | 0.077 | 0.0008 | 0.078 | 0.0006 |
| 0.10-0.12 | 0.089 | 0.0010 | 11 | 0.093 | 0.0013 | 5 | 0.092 | 0.0007 | 9 | 0.090 | 0.0009 | 0.093 | 0.0005 |
| 0.12-0.14 | 0.099 | 0.0017 | 12 | 0.104 | 0.0010 | 5 | 0.102 | 0.0020 | 10 | 0.100 | 0.0013 | 0.103 | 0.0005 |
| 0.14-0.16 | 0.108 | 0.0026 | 10 | 0.110 | 0.0013 | 5 | 0.112 | 0.0019 | 10 | 0.109 | 0.0018 | 0.111 | 0.0008 |
| 0.16-0.18 | 0.117 | 0.0029 | 9 | 0.117 | 0.0010 | 5 | 0.122 | 0.0018 | 9 | 0.117 | 0.0018 | 0.120 | 0.0007 |
| 0.18-0.20 | 0.124 | 0.0033 | 10 | 0.125 | 0.0008 | 5 | 0.133 | 0.0021 | 8 | 0.125 | 0.0022 | 0.130 | 0.0006 |
| 0.20-0.22 | 0.130 | 0.0021 | 6 | 0.135 | 0.0023 | 4 | 0.142 | 0.0024 | 7 | 0.132 | 0.0017 | 0.140 | 0.0006 |
| 0.22-0.24 | 0.137 | 0.0027 | 5 | 0.146 | 0.0023 | 3 | 0.161 |  | 1 | 0.140 | 0.0025 | 0.150 | 0.0006 |
| 0.24-0.26 | 0.148 | 0.0076 | 3 | 0.154 |  | 1 | 0.173 |  | 1 | 0.150 | 0.0056 | 0.164 | 0.0014 |
| 0.26-0.28 | 0.143 |  | 1 |  |  |  | 0.183 |  | 1 | 0.143 |  | 0.183 |  |
| 0.28-0.30 | 0.155 |  | 1 |  |  |  |  |  |  | 0.155 |  |  |  |
| 0.30-0.32 | 0.161 |  | 1 |  |  |  |  |  |  | 0.161 |  |  |  |

Remark. I – Indomalaya and Afrothropic realms. II – Holarctic, Indomalaya and Afrothropic realms, III – Holarctic (Nearctic and/or Palearctic) realm.
