# Supplementary material for: Transition–Transversion Bias at the CYTB Gene Level in the Order Cypriniformes (Actinopterygii) as Evidence for the Influence of Metabolic Rate on Molecular Evolutionary Rate
Source: Ecol Evol. 2026 Jun 29;16(7):e73905. doi: 10.1002/ece3.73905 (PMC13314720; doi:10.1002/ece3.73905)
Supplement: Supplementary file 4 — Table S4: Student's t‐tests (t) and number of degrees of freedom (df) as well results of two‐way ANOVA (F) obtained from comparisons of mean transition frequencies of nucleotide substitution classes of Cypriniformes subfamilies/families of different bioclimatic zones. [file ECE3-16-e73905-s004.docx]

Table S4. Student’s t-tests (t) and number of degrees of freedom (df) as well results of two-way ANOVA (F) obtained from comparisons of mean transition frequencies of nucleotide substitution classes of Cypriniformes subfamilies/families of different bioclimatic zones

| Substution  classes | Comparison options | | | | | | | |
| --- | --- | --- | --- | --- | --- | --- | --- | --- |
|  | I-II | | II-III | | I-III | | (I+II)-III | I-(II+III) |
|  | t | df | t | df | t | df | t | t |
| 0-0.02 | -0.24 | 16 | 0.30 | 12 | 0.00 | 20 | 0.08 | -0.09 |
| 0.02-0.04 | 0.20 | 13 | 0.18 | 13 | 0.29 | 18 | 0.31 | 0.26 |
| 0.04-0.06 | 0.01 | 16 | 1.06 | 12 | 0.69 | 20 | 0.86 | 0.48 |
| 0.06-0.08 | -1.97 | 15 | 0.43 | 13 | -1.79 | 20 | -1.26 | -2.12 |
| 0.08-0.10 | -2.74 | 15 | 2.00 | 13 | -1.01 | 20 | -0.26 | -1.68 |
| 0.10-0.12 | -2.30 | 14 | 0.32 | 12 | -2.76 | 18 | -1.89 | -2.99 |
| 0.12-0.14 | -2.55 | 15 | 0.89 | 13 | -1.11 | 20 | -0.60 | -1.66 |
| 0.14-0.16 | -0.72 | 13 | -0.81 | 13 | -1.24 | 18 | -1.27 | -1.15 |
| 0.16-0.18 | -0.18 | 12 | -2.57 | 12 | -1.72 | 16 | -2.19 | -1.24 |
| 0.18-0.20 | -0.32 | 13 | -3.18 | 11 | -2.12 | 16 | -2.62 | -1.50 |
| 0.20-0.22 | -1.66 | 8 | -1.98 | 9 | -3.69 | 11 | -3.31 | -3.27 |
| 0.22-0.24 | -2.66 | 6 |  |  |  |  | -8.51 |  |
| ANOVA | | | | | | | | |
| F | 7,2 | | 2,8 | | 15,05 | | 16,5 | 13,2 |
| df_1_, df_2_ | 1, 156 | | 1, 134 | | 1, 198 | | 1, 252 | 1, 252 |

Remarks. Significant meanings are highlighted in color.
