# Supplementary material for: Transition–Transversion Bias at the CYTB Gene Level in the Order Cypriniformes (Actinopterygii) as Evidence for the Influence of Metabolic Rate on Molecular Evolutionary Rate
Source: Ecol Evol. 2026 Jun 29;16(7):e73905. doi: 10.1002/ece3.73905 (PMC13314720; doi:10.1002/ece3.73905)
Supplement: Supplementary file 5 — Table S5: Means (M) and standard errors (SE) of transversion frequencies within classes of nucleotide substitutions and number of subfamilies/families (N) within bioclimatic zones. [file ECE3-16-e73905-s001.docx]

Table S5. Means (M) and standard errors (SE) of transversion frequencies within classes of nucleotide substitutions and number of subfamilies/families (N) within bioclimatic zones

| Substitu-tion  classes | Bioclimatic zones | | | | | | | | | | | | |
| --- | --- | --- | --- | --- | --- | --- | --- | --- | --- | --- | --- | --- | --- |
|  | I | | | II | | | III | | | I + II | | II + III | |
|  | M | SE | N | M | SE | N | M | SE | N | M | SE | M | SE |
| 0-0.02 | 0.001 | 0.0002 | 13 | 0.001 | 0.0001 | 5 | 0.001 | 0.0002 | 10 | 0.001 | 0.0001 | 0.001 | 0.0001 |
| 0.02-0.04 | 0.003 | 0.0004 | 10 | 0.003 | 0.0006 | 5 | 0.004 | 0.0002 | 10 | 0.003 | 0.0003 | 0.003 | 0.0003 |
| 0.04-0.06 | 0.006 | 0.0004 | 13 | 0.005 | 0.0007 | 5 | 0.006 | 0.0005 | 9 | 0.006 | 0.0004 | 0.005 | 0.0004 |
| 0.06-0.08 | 0.008 | 0.0006 | 12 | 0.007 | 0.0010 | 5 | 0.009 | 0.0008 | 10 | 0.008 | 0.0005 | 0.008 | 0.0006 |
| 0.08-0.10 | 0.015 | 0.0007 | 12 | 0.012 | 0.0013 | 5 | 0.013 | 0.0009 | 10 | 0.014 | 0.0006 | 0.013 | 0.0007 |
| 0.10-0.12 | 0.023 | 0.0009 | 11 | 0.019 | 0.0014 | 5 | 0.019 | 0.0008 | 9 | 0.022 | 0.0009 | 0.019 | 0.0007 |
| 0.12-0.14 | 0.032 | 0.0021 | 12 | 0.026 | 0.0015 | 5 | 0.029 | 0.0020 | 10 | 0.030 | 0.0016 | 0.028 | 0.0015 |
| 0.14-0.16 | 0.043 | 0.0026 | 10 | 0.041 | 0.0021 | 5 | 0.039 | 0.0022 | 10 | 0.042 | 0.0018 | 0.040 | 0.0016 |
| 0.16-0.18 | 0.053 | 0.0023 | 9 | 0.053 | 0.0012 | 5 | 0.046 | 0.0028 | 9 | 0.053 | 0.0015 | 0.049 | 0.0020 |
| 0.18-0.20 | 0.064 | 0.0032 | 10 | 0.062 | 0.0012 | 5 | 0.055 | 0.0022 | 8 | 0.063 | 0.0021 | 0.057 | 0.0017 |
| 0.20-0.22 | 0.079 | 0.0031 | 6 | 0.071 | 0.0008 | 4 | 0.062 | 0.0020 | 7 | 0.076 | 0.0022 | 0.065 | 0.0019 |
| 0.22-0.24 | 0.091 | 0.0026 | 5 | 0.080 | 0.0018 | 3 | 0.072 |  | 1 | 0.087 | 0.0026 | 0.078 | 0.0023 |
| 0.24-0.26 | 0.103 | 0.0092 | 3 |  |  |  | 0.076 |  | 1 | 0.099 | 0.0073 | 0.083 | 0.0065 |
| 026-0,28 | 0.122 |  | 1 |  |  |  | 0.082 |  | 1 | 0.122 |  | 0.082 |  |
| 0,28-0,30 | 0.141 |  | 1 |  |  |  |  |  |  | 0.141 |  |  |  |
| 0,30-0,32 | 0.150 |  | 1 |  |  |  |  |  |  | 0.150 |  |  |  |

Remark: I – Indomalaya and Afrothropic realms, II – Holarctic, Indomalaya and Afrothropic realms, III – Holarctic (Nearctic and/or Palearctic) realm.
