# Supplementary material for: Transition–Transversion Bias at the CYTB Gene Level in the Order Cypriniformes (Actinopterygii) as Evidence for the Influence of Metabolic Rate on Molecular Evolutionary Rate
Source: Ecol Evol. 2026 Jun 29;16(7):e73905. doi: 10.1002/ece3.73905 (PMC13314720; doi:10.1002/ece3.73905)
Supplement: Supplementary file 6 — Table S6: Student's t‐tests (t) and number of degrees of freedom (df) as well results of two‐way ANOVA (F) obtained from comparisons of mean transversion frequencies of nucleotide substitution classes of Cypriniformes subfamilies/families of different bioclimatic zones. [file ECE3-16-e73905-s011.docx]

Table S6. Student’s t-tests (t) and number of degrees of freedom (df) as well results of two-way ANOVA (F) obtained from comparisons of mean transversion frequencies of nucleotide substitution classes of Cypriniformes subfamilies/families of different bioclimatic zones

| Substitu-  tion  classes | Comparison options | | | | | | | |
| --- | --- | --- | --- | --- | --- | --- | --- | --- |
|  | I-II | | II-III | | I-III | | (I+II)-III | I-(II+III) |
|  | t | df | t | df | t | df | t | t |
| 0-0.02 | -0.97 | 16 | -0.91 | 21 | -1.51 | 13 | -1.50 | -1.44 |
| 0.02-0.04 | 0.27 | 13 | -1.56 | 18 | -1.80 | 13 | -2.22 | -1.01 |
| 0.04-0.06 | 1.41 | 16 | -0.60 | 20 | 0.95 | 12 | 0.47 | 1.43 |
| 0.06-0.08 | 0.85 | 15 | -0.92 | 20 | -0.21 | 13 | -0.52 | 0.22 |
| 0.08-0.10 | 1.61 | 15 | -0.15 | 20 | 1.88 | 13 | 1.29 | 2.24 |
| 0.10-0.12 | 2.72 | 14 | -0.22 | 18 | 3.42 | 12 | 2.27 | 3.75 |
| 0.12-0.14 | 2.27 | 15 | -1.27 | 20 | 0.88 | 13 | 0.33 | 1.43 |
| 0.14-0.16 | 0.46 | 13 | 0.67 | 18 | 1.06 | 13 | 1.08 | 0.95 |
| 0.16-0.18 | -0.06 | 12 | 2.25 | 16 | 1.85 | 12 | 2.13 | 1.38 |
| 0.18-0.20 | 0.71 | 13 | 2.88 | 16 | 2.51 | 11 | 2.89 | 1.91 |
| 0.20-0.22 | 2.39 | 8 | 4.40 | 11 | 4.62 | 9 | 4.71 | 3.74 |
| 0.22-0.24 | 3.54 | 6 |  |  |  |  |  | 3.75 |
| ANOVA | | | | | | | | |
| F | 14.9 |  | 4.6 |  | 25.9 |  | 22.8 | 25.5 |
| df_1_, df_2_ | 1, 156 |  | 1, 134 |  | 1, 198 |  | 1, 252 | 1, 252 |

Remarks. Significant meanings are highlighted in color.
