# Supplementary material for: Transition–Transversion Bias at the CYTB Gene Level in the Order Cypriniformes (Actinopterygii) as Evidence for the Influence of Metabolic Rate on Molecular Evolutionary Rate
Source: Ecol Evol. 2026 Jun 29;16(7):e73905. doi: 10.1002/ece3.73905 (PMC13314720; doi:10.1002/ece3.73905)
Supplement: Supplementary file 7 — Table S7: Means (M) and standard errors (SE) of ts/tv index within classes of nucleotide substitutions and number of subfamilies/families (N) within bioclimatic zones. [file ECE3-16-e73905-s008.docx]

Table S7. Means (M) and standard errors (SE) of ts/tv index within classes of nucleotide substitutions and number of subfamilies/families (N) within bioclimatic zones

| Substitu-tion  classes | Bioclimatic zones | | | | | | | | | | | | |
| --- | --- | --- | --- | --- | --- | --- | --- | --- | --- | --- | --- | --- | --- |
|  | I | | | II | | | III | | | I + II | | II + III | |
|  | M | SE | N | M | SE | N | M | SE | N | M | SE | M | SE |
| 0-0.02 | 0.784 | 0.047 | 13 | 0.745 | 0.028 | 5 | 0.706 | 0.029 | 10 | 0.773 | 0.035 | 0.719 | 0.021 |
| 0.02-0.04 | 0.819 | 0.014 | 10 | 0.828 | 0.038 | 5 | 0.763 | 0.011 | 10 | 0.822 | 0.015 | 0.785 | 0.016 |
| 0.04-0.06 | 0.759 | 0.015 | 13 | 0.802 | 0.029 | 5 | 0.778 | 0.019 | 9 | 0.771 | 0.014 | 0.786 | 0.016 |
| 0.06-0.08 | 0.758 | 0.014 | 12 | 0.791 | 0.027 | 5 | 0.760 | 0.021 | 10 | 0.768 | 0.013 | 0.771 | 0.017 |
| 0.08-0.10 | 0.676 | 0.014 | 12 | 0.731 | 0.026 | 5 | 0.721 | 0.018 | 10 | 0.692 | 0.014 | 0.724 | 0.014 |
| 0.10-0.12 | 0.589 | 0.015 | 11 | 0.667 | 0.025 | 5 | 0.660 | 0.014 | 9 | 0.613 | 0.016 | 0.662 | 0.012 |
| 0.12-0.14 | 0.517 | 0.030 | 12 | 0.602 | 0.021 | 5 | 0.557 | 0.030 | 10 | 0.542 | 0.024 | 0.572 | 0.022 |
| 0.14-0.16 | 0.434 | 0.035 | 10 | 0.458 | 0.025 | 5 | 0.484 | 0.028 | 10 | 0.442 | 0.024 | 0.475 | 0.020 |
| 0.16-0.18 | 0.375 | 0.029 | 9 | 0.376 | 0.013 | 5 | 0.453 | 0.030 | 9 | 0.375 | 0.019 | 0.425 | 0.022 |
| 0.18-0.20 | 0.319 | 0.033 | 10 | 0.340 | 0.010 | 5 | 0.417 | 0.023 | 8 | 0.326 | 0.022 | 0.387 | 0.018 |
| 0.20-0.22 | 0.248 | 0.025 | 6 | 0.312 | 0.012 | 4 | 0.395 | 0.019 | 7 | 0.273 | 0.019 | 0.365 | 0.019 |
| 0.22-0.24 | 0.202 | 0.022 | 5 | 0.295 | 0.017 | 3 | 0.384 |  | 1 | 0.237 | 0.022 | 0.317 | 0.026 |
| 0.24-0.26 | 0.182 | 0.068 | 3 | 0.266 |  | 1 | 0.388 |  | 1 | 0.203 | 0.052 | 0.327 | 0.061 |
| 0.26-0.28 | 0.076 |  | 1 |  |  |  | 0.384 |  | 1 | 0.076 |  | 0.384 |  |
| 0.28-0.30 | 0.047 |  | 1 |  |  |  |  |  |  | 0.047 |  |  |  |
| 0.30-0.32 | 0.034 |  | 1 |  |  |  |  |  |  | 0.034 |  |  |  |

Remark: I – Indomalaya and Afrothropic realms, II – Holarctic, Indomalaya and Afrothropic realms, III – Holarctic(Palearctic and/or Nearctic) realm.
