# Supplementary material for: Transition–Transversion Bias at the CYTB Gene Level in the Order Cypriniformes (Actinopterygii) as Evidence for the Influence of Metabolic Rate on Molecular Evolutionary Rate
Source: Ecol Evol. 2026 Jun 29;16(7):e73905. doi: 10.1002/ece3.73905 (PMC13314720; doi:10.1002/ece3.73905)
Supplement: Supplementary file 8 — Table S8: Student's t‐tests (t) and number of degree of freedoms (df) as well results of two‐way ANOVA (F) obtained from comparisons of mean transversion frequencies of nucleotide substitution classes of Cypriniformes subfamilies/families of different bioclimatic zones. [file ECE3-16-e73905-s005.docx]

Table S8. Student’s t-tests (t) and number of degree of freedoms (df) as well results of two-way ANOVA (F) obtained from comparisons of mean transversion frequencies of nucleotide substitution classes of Cypriniformes subfamilies/families of different bioclimatic zones

| Substitu-tion  classes | Comparison options | | | | | | | |
| --- | --- | --- | --- | --- | --- | --- | --- | --- |
|  | I-II | | II-III | | I-III | | (I+II)-III | I-(II+III) |
|  | t_st_ | N | t_st_ | N | t_st_ | N | t_st_ | t_st_ |
| 0-0.02 | 0.71 | 16 | 0.97 | 21 | 1.41 | 13 | 1.49 | 1.25 |
| 0.02-0.04 | -0.21 | 13 | 1.63 | 18 | 3.12 | 13 | 3.17 | 1.61 |
| 0.04-0.06 | -1.29 | 16 | 0.67 | 20 | -0.76 | 12 | -0.30 | -1.23 |
| 0.06-0.08 | -1.08 | 15 | 0.90 | 20 | -0.08 | 13 | 0.31 | -0.56 |
| 0.08-0.10 | -1.86 | 15 | 0.32 | 20 | -1.98 | 13 | -1.29 | -2.41 |
| 0.10-0.12 | -2.71 | 14 | 0.23 | 18 | -3.54 | 12 | -2.29 | -3.87 |
| 0.12-0.14 | -2.30 | 15 | 1.22 | 20 | -0.93 | 13 | -0.38 | -1.48 |
| 0.14-0.16 | -0.54 | 13 | -0.72 | 18 | -1.12 | 13 | -1.15 | -1.02 |
| 0.16-0.18 | -0.05 | 12 | -2.33 | 16 | -1.87 | 12 | -2.19 | -1.39 |
| 0.18-0.20 | -0.60 | 13 | -3.14 | 16 | -2.46 | 11 | -2.90 | -1.83 |
| 0.20-0.22 | -2.28 | 8 | -3.45 | 11 | -4.49 | 9 | -4.36 | -3.73 |
| 0.22-0.24 | -3.35 | 6 | 0.97 | 3 |  |  |  | -3.43 |
| 0.24-0.26 |  |  |  |  |  |  |  | -1.60 |
|  | | | | | | | | |
| F | 7,7 | | 0,02 | | 8,0 | | 5,0 | 10,8 |
| df_1_, df_2_ | 1, 156 | | 1, 134 | | 1, 198 | | 1, 252 | 1, 252 |

Remarks. Significant meanings are highlighted in color.
