# Supplementary material for: Transition–Transversion Bias at the CYTB Gene Level in the Order Cypriniformes (Actinopterygii) as Evidence for the Influence of Metabolic Rate on Molecular Evolutionary Rate
Source: Ecol Evol. 2026 Jun 29;16(7):e73905. doi: 10.1002/ece3.73905 (PMC13314720; doi:10.1002/ece3.73905)
Supplement: Supplementary file 9 — Table S9: Means (M), standard errors (SE) of transition (Ts), transversion (Tv) frequencies, ts/tv indices (Ts/Tv) and number of subfamilies/families (N) for each class of nucleotide substitutions in small body sized and large body sized subfamilies/families of the Cypriniformes and its comparisons by Student's t‐test and two‐way ANOVA. [file ECE3-16-e73905-s003.docx]

Таble S9. Means (M), standard errors (SE) of transition (Ts), transversion (Tv) frequencies, ts/tv indices (Ts/Tv) and number of subfamilies/families (N) for each class of nucleotide substitutions in small body sized and large body sized subfamilies/families of the Cypriniformes and its comparisons by Student’s t-test and two-way ANOVA

| Substitution  level | Small body sized subfamilies/families | | | | | | | Large body sized subfamilies/families | | | | | | | | t | | |
| --- | --- | --- | --- | --- | --- | --- | --- | --- | --- | --- | --- | --- | --- | --- | --- | --- | --- | --- |
|  | Ts | | Tv | | Ts/Tv | | N | Ts | | Tv | | | Ts/Tv | | N |  |  |  |
|  | M | SE | M | SE | M | SE |  | M | SE | M | SE | | M | SE |  | Ts | Tv | Ts/Tv |
| 0-0.02 | 0.007 | 0.001 | 0.001 | 0.0002 | 0.733 | 0.038 | 17 | 0.008 | 0.001 | 0.001 | 0.0001 | | 0.774 | 0.027 | 11 | -1.28 | 0.53 | -0.88 |
| 0.02-0.04 | 0.029 | 0.002 | 0.003 | 0.0003 | 0.793 | 0.043 | 14 | 0.028 | 0.001 | 0.003 | 0.0004 | | 0.805 | 0.016 | 11 | 0.07 | 0.31 | -0.47 |
| 0.04-0.06 | 0.045 | 0.001 | 0.007 | 0.0003 | 0.746 | 0.011 | 17 | 0.046 | 0.001 | 0.005 | 0.0004 | | 0.820 | 0.019 | 10 | -0.10 | 4.18 | -4.15 |
| 0.06-0.08 | 0.061 | 0.001 | 0.009 | 0.001 | 0.752 | 0.014 | 16 | 0.064 | 0.001 | 0.008 | 0.001 | | 0.785 | 0.016 | 11 | -2.18 | 1.30 | -1.52 |
| 0.08-0.10 | 0.077 | 0.001 | 0.014 | 0.001 | 0.686 | 0.013 | 16 | 0.079 | 0.001 | 0.013 | 0.001 | | 0.726 | 0.022 | 11 | -1.75 | 1.51 | -1.72 |
| 0.10-0.12 | 0.091 | 0.001 | 0.021 | 0.001 | 0.622 | 0.013 | 15 | 0.092 | 0.001 | 0.020 | 0.001 | | 0.641 | 0.024 | 10 | -0.82 | 0.67 | -0.71 |
| 0.12-0.14 | 0.100 | 0.002 | 0.031 | 0.002 | 0.528 | 0.028 | 16 | 0.103 | 0.001 | 0.028 | 0.002 | | 0.576 | 0.024 | 11 | -1.74 | 1.28 | -1.37 |
| 0.14-0.16 | 0.108 | 0.002 | 0.044 | 0.002 | 0.424 | 0.028 | 14 | 0.113 | 0.001 | 0.037 | 0.001 | | 0.504 | 0.015 | 11 | -2.24 | 2.63 | -2.56 |
| 0.16-0.18 | 0.116 | 0.002 | 0.053 | 0.002 | 0.372 | 0.019 | 13 | 0.122 | 0.002 | 0.047 | 0.002 | | 0.449 | 0.026 | 10 | -2.13 | 2.26 | -2.26 |
| 0.18-0.20 | 0.125 | 0.002 | 0.063 | 0.002 | 0.330 | 0.018 | 14 | 0.131 | 0.003 | 0.056 | 0.003 | | 0.401 | 0.029 | 9 | -1.47 | 2.02 | -1.89 |
| 0.20-0.22 | 0.135 | 0.002 | 0.071 | 0.003 | 0.314 | 0.024 | 11 | 0.139 | 0.003 | 0.068 | 0.004 | | 0.341 | 0.033 | 6 | -0.92 | 0.47 | -0.62 |
| 0.22-0.24 | 0.142 | 0.004 | 0.087 | 0.003 | 0.240 | 0.028 | 7 | 0.147 | 0.004 | 0.079 | 0.003 | | 0.301 | 0.027 | 2 | -0.93 | 1.85 | -1.49 |
| 0.24-0.26 | 0.154 | 0.006 | 0.095 | 0.007 | 0.240 | 0.047 | 5 |  |  |  |  | |  |  |  | ANOVA | | |
| 0.26-0.28 | 0.163 | 0.020 | 0.102 | 0.020 | 0.230 | 0.084 | 2 |  |  |  |  | |  |  |  | 16.4 | 21.8 | 24.4 |
| 0.28-0.30 | 0.155 |  | 0.141 |  | 0.047 |  | 1 |  |  |  | | |  |  |  | df_1_ = 1, df_2_ = 259 | | |
| 0.30-0.32 | 0.161 |  | 0.150 |  | 0.034 |  | 1 |  |  |  | |  |  |  | |  | | |

Remarks. Significant meanings are highlighted in color.
