# Supplementary material for: Transition–Transversion Bias at the CYTB Gene Level in the Order Cypriniformes (Actinopterygii) as Evidence for the Influence of Metabolic Rate on Molecular Evolutionary Rate
Source: Ecol Evol. 2026 Jun 29;16(7):e73905. doi: 10.1002/ece3.73905 (PMC13314720; doi:10.1002/ece3.73905)
Supplement: Supplementary file 10 — Table S10: Means (M). standard errors (SE). of transition (Ts). transversion (Tv) frequencies. ts/tv indices (Ts/Tv) and number of subfamilies/families (N) for each class of nucleotide substitutions in small‐body sized and large‐body sized suborders of the Cypriniformes and its comparisons by Student's t‐test and two‐way ANOVA. [file ECE3-16-e73905-s009.docx]

Table S10. Means (M). standard errors (SE). of transition (Ts). transversion (Tv) frequencies. ts/tv indices (Ts/Tv) and number of subfamilies/families (N) for each class of nucleotide substitutions in small-body sized and large-body sized suborders of the Cypriniformes and its comparisons by Student’s t-test and two-way ANOVA

| Substitution  level | Small body sized suborders | | | | | | | Large body sized suborders | | | | | | | t | | |
| --- | --- | --- | --- | --- | --- | --- | --- | --- | --- | --- | --- | --- | --- | --- | --- | --- | --- |
|  | Ts | | Tv | | Ts/Tv | | N | Ts | | Tv | | Ts/Tv | | N |  |  |  |
|  | M | SE | M | SE | M | SE |  | M | SE | M | SE | M | SE |  | Ts | Tv | Ts/Tv |
| 0-0.02 | 0.007 | 0.0005 | 0.001 | 0.0003 | 0.748 | 0.055 | 5 | 0.007 | 0.001 | 0.001 | 0.0001 | 0.752 | 0.029 | 23 | 0.32 | -0.06 | 0.06 |
| 0.02-0.04 | 0.027 | 0.001 | 0.003 | 0.001 | 0.817 | 0.029 | 5 | 0.029 | 0.001 | 0.005 | 0.002 | 0.756 | 0.029 | 20 | 1.30 | 1.34 | -1.50 |
| 0.04-0.06 | 0.046 | 0.001 | 0.007 | 0.000 | 0.741 | 0.019 | 6 | 0.045 | 0.001 | 0.006 | 0.0003 | 0.778 | 0.012 | 21 | -0.50 | -2.15 | 1.64 |
| 0.06-0.08 | 0.060 | 0.002 | 0.009 | 0.001 | 0.732 | 0.011 | 5 | 0.062 | 0.001 | 0.008 | 0.001 | 0.763 | 0.014 | 22 | 0.92 | -1.27 | 1.78 |
| 0.08-0.10 | 0.078 | 0.001 | 0.014 | 0.001 | 0.689 | 0.015 | 6 | 0.077 | 0.001 | 0.014 | 0.001 | 0.700 | 0.014 | 21 | -0.41 | -0.66 | 0.55 |
| 0.10-0.12 | 0.089 | 0.001 | 0.021 | 0.001 | 0.622 | 0.023 | 6 | 0.092 | 0.001 | 0.021 | 0.001 | 0.629 | 0.013 | 19 | 1.40 | 0.01 | 0.27 |
| 0.12-0.14 | 0.098 | 0.003 | 0.034 | 0.004 | 0.485 | 0.056 | 5 | 0.101 | 0.001 | 0.029 | 0.001 | 0.558 | 0.018 | 22 | 1.08 | -1.27 | 1.23 |
| 0.14-0.16 | 0.103 | 0.004 | 0.048 | 0.004 | 0.367 | 0.051 | 5 | 0.111 | 0.001 | 0.040 | 0.001 | 0.476 | 0.017 | 20 | 2.09 | -1.99 | 2.03 |
| 0.16-0.18 | 0.113 | 0.003 | 0.056 | 0.002 | 0.339 | 0.030 | 5 | 0.121 | 0.001 | 0.049 | 0.002 | 0.422 | 0.020 | 18 | 2.45 | -2.21 | 2.32 |
| 0.18-0.20 | 0.122 | 0.004 | 0.063 | 0.003 | 0.315 | 0.032 | 5 | 0.129 | 0.002 | 0.059 | 0.002 | 0.372 | 0.021 | 18 | 1.80 | -1.19 | 1.49 |
| 0.20-0.22 | 0.132 | 0.004 | 0.072 | 0.0003 | 0.294 | 0.014 | 2 | 0.137 | 0.002 | 0.069 | 0.003 | 0.333 | 0.023 | 15 | 1.16 | -1.27 | 0.06 |
| 0.22-0.24 | 0.145 |  | 0.081 |  | 0.282 |  | 1 | 0.145 | 0.004 | 0.084 | 0.004 | 0.264 | 0.033 | 8 | ANOVA | | |
| 0.24-0.26 | 0.154 |  | 0.089 |  | 0.266 |  | 1 | 0.158 | 0.007 | 0.092 | 0.008 | 0.264 | 0.063 | 5 | Ts | Tv | Ts/Tv |
| 0.26-0.28 |  |  |  |  |  |  |  | 0.170 | 0.014 | 0.095 | 0.014 | 0.282 | 0.103 | 3 | 11.8 | 9.4 | 6.8 |
| 0.28-0.30 |  |  |  |  |  |  |  | 0.155 |  | 0.141 |  | 0.047 |  | 1 | df_1_ = 1, df_2_ = 252 | | |
| 0.30-0.32 |  |  |  |  |  |  |  | 0.161 |  | 0.150 |  | 0.034 |  | 1 |  |  |  |

Remarks. Significant differences are highlighted in color.

.
