# Supplementary material for: Transition–Transversion Bias at the CYTB Gene Level in the Order Cypriniformes (Actinopterygii) as Evidence for the Influence of Metabolic Rate on Molecular Evolutionary Rate
Source: Ecol Evol. 2026 Jun 29;16(7):e73905. doi: 10.1002/ece3.73905 (PMC13314720; doi:10.1002/ece3.73905)
Supplement: Supplementary file 11 — Table S11: Means (M) and standard errors (SE) of ts/tv index within classes of nucleotide substitutions and number of analyzed of subfamilies/families (N) of climatic zone types and body sized groups combined and its comparisons by Student's t‐test and two‐way ANOVA. [file ECE3-16-e73905-s006.docx]

Table S11. Means (M) and standard errors (SE) of ts/tv index within classes of nucleotide substitutions and number of analyzed of subfamilies/families (N) of climatic zone types and body sized groups combined and its comparisons by Student’s t-test and two-way ANOVA

| Substitu-tion  classes | Small sized  subfamilies/families of Afrotropic and Indo-Malaya realms  (Group A) | | | Small body-sized Holarctic and large body-sized Afrotropic and Indo-Malaya  subfamilies/families as well as subfamilies/families of mixes ranges  (Group B) | | | Large sized Holarctic  subfamilies/families  (Group C) | | | t | | | |
| --- | --- | --- | --- | --- | --- | --- | --- | --- | --- | --- | --- | --- | --- |
|  | M | SE | N | M | SE | N | M | SE | N | A-B | A-C | | B-C |
| 0.02-0.04 | 0.751 | 0.058 | 10 | 0.757 | 0.029 | 14 | 0.715 | 0.033 | 4 | -0.09 | 0.55 | | 0.97 |
| 0.04-0.06 | 0.818 | 0.020 | 7 | 0.800 | 0.016 | 14 | 0.759 | 0.025 | 4 | 0.56 | 1.84 | | 1.38 |
| 0.06-0.08 | 0.745 | 0.017 | 10 | 0.782 | 0.015 | 14 | 0.828 | 0.027 | 3 | -1.16 | -2.61 | | -1.50 |
| 0.08-0.10 | 0.758 | 0.019 | 9 | 0.763 | 0.016 | 14 | 0.786 | 0.032 | 4 | -0.13 | -0.75 | | -0.64 |
| 0.10-0.12 | 0.675 | 0.014 | 9 | 0.704 | 0.016 | 14 | 0.759 | 0.023 | 4 | -1.07 | -3.10 | | -1.97 |
| 0.12-0.14 | 0.596 | 0.017 | 8 | 0.637 | 0.015 | 14 | 0.688 | 0.034 | 3 | -1.06 | -2.43 | | -1.41 |
| 0.14-0.16 | 0.515 | 0.039 | 9 | 0.556 | 0.021 | 14 | 0.592 | 0.048 | 4 | -0.68 | -1.26 | | -0.68 |
| 0.16-0.18 | 0.410 | 0.043 | 7 | 0.461 | 0.021 | 14 | 0.537 | 0.018 | 4 | -1.09 | -2.71 | | -2.81 |
| 0.18-0.20 | 0.362 | 0.032 | 7 | 0.391 | 0.015 | 12 | 0.524 | 0.038 | 4 | -0.59 | -3.28 | | -3.29 |
| 0.20-0.22 | 0.297 | 0.027 | 8 | 0.369 | 0.021 | 12 | 0.474 | 0.028 | 3 | -1.82 | -4.52 | | -2.99 |
| 0.22-0.24 | 0.253 | 0.030 | 5 | 0.335 | 0.020 | 10 | 0.443 | 0.004 | 2 | -2.68 | -6.22 | | -5.24 |
| 0.24-0.26 | 0.202 | 0.022 | 5 | 0.317 | 0.026 | 4 |  |  |  | ANOVA | | | |
| 0.26-0.28 | 0.182 | 0.068 | 3 | 0.327 | 0.061 | 2 |  |  |  | F | | | |
| 0.28-0.30 | 0.076 |  | 1 | 0.384 | 0.029 | 1 |  |  |  | 9.0 | 21.1 | 13.9 | |
| 0.30-0.32 | 0.047 |  | 1 |  |  |  |  |  |  | df_1_, df_2_ | | | |
| 0.32-0.34 | 0.034 |  | 1 |  |  |  |  |  |  | 1, 213 | 1, 106 | | 1, 163 |

Remarks. Significant differences are highlighted in color.
